# The co-expression of the depolarizing and hyperpolarizing mechanosensitive ion channels in mammalian retinal neurons

**DOI:** 10.3389/fmed.2024.1463898

**Published:** 2024-11-13

**Authors:** Vivian Y. Pang, Zhuo Yang, Samuel M. Wu, Ji-Jie Pang

**Affiliations:** Department of Ophthalmology, Baylor College of Medicine, Houston, TX, United States

**Keywords:** TRAAK, BK, TRPV2, TRPV4, ENaC, immunocytochemistry, confocal microscopy, whole-cell patch-clamp

## Abstract

**Introduction:**

The elevation of the intraocular and extraocular pressures is associated with various visual conditions, including glaucoma and traumatic retinal injury. The retina expresses mechanosensitive channels (MSCs), but the role of MSCs in retinal physiology and pathologies has been unclear.

**Methods:**

Using immunocytochemistry, confocal microscopy, and patch-clamp recording techniques, we studied the co-expression of K^+^-permeable (K-MSCs) TRAAK and big potassium channel BK with the epithelial sodium channel ENaC and transient receptor potential channel vanilloid TPRV4 and TRPV2 favorably permeable to Ca^2+^ than Na^+^ (together named N-MSCs), and TRPV4 activity in the mouse retina.

**Results:**

TRAAK immunoreactivity (IR) was mainly located in Müller cells. Photoreceptor outer segments (OSs) expressed BK and ENaCα intensively and TRAAK, TRPV2, and TRPV4 weakly. Somas and axons of retinal ganglion cells (RGCs) retrograde-identified clearly expressed ENaCα, TRPV4, and TRPV2 but lacked TRAAK and BK. Rod bipolar cells (RBCs) showed TRPV4-IR in somas and BK-IR in axonal globules. Horizontal cells were BK-negative, and some cone BCs lacked TRPV4-IR. TRPV4 agonist depolarized RGCs, enhanced spontaneous spikes and excitatory postsynaptic currents, reduced the visual signal reliability (*VSR = 1-noise/signal*) by ~50%, and resulted in ATP crisis, which could inactivate voltage-gated sodium channels in RGCs.

**Conclusion:**

Individual neurons co-express hyperpolarizing K-MSCs with depolarizing N-MSCs to counterbalance the pressure-induced excitation, and the level of K-MSCs relative to N-MSCs (*R_K/N_* ratio) is balanced in the outer retina but low in RGCs, bringing out novel determinants for the pressure vulnerability of retinal neurons and new targets for clinical interventions.

## Introduction

Retinal neurons are vulnerable to pressure stresses in glaucoma (1, 2), traumatic retinal injury (TRI) (3–6), and other conditions (7), and all eukaryocytes express mechanosensitive ion channels (MSCs) to cope with changes in pressure, osmolarity, temperature, shape, and volume (8–10). Multiple types of MSCs are present in retinal neurons, but their role has been unclear.

MSCs vary by structure, ion permeability and selectivity, modulator, and other characteristics. Some MSCs may mediate membrane depolarization, such as Na^+^/Ca^2+^-permeable transient receptor potential channel vanilloid (TRPVs) and Na^+^-permeable epithelial sodium channel (ENaC, SCNN1), and TRPV activation may lead to neuronal excitotoxicity (11–15) and involves the injury to retinal ganglion cells (RGCs) in glaucoma models (16–18). However, not all MSCs mediate depolarizing Ca^2+^ or Na^+^ currents. In this report, we classified MSCs into two categories based on their permeability and effects on resting membrane potential: the hyperpolarizing K-MSCs permeable to K^+^ and the depolarizing N-MSCs selective for Na^+^ or nonselective for cations, proposing a potential neuroprotective role of K-MSCs.

Vertebrate retinal rods and the outer plexiform layer (OPL) express N-MSCs like TRPV1 (19), TRPV2 (20–22), and TRPV4 (22, 23), as well as K-MSCs like the big potassium channel (24–26) (BK, also known as the calcium-and voltage-gated large conductance potassium channel, Maxi-K, KCNMA1, Slo1, Kca1.1, and stretch-activated potassium channels) and the two-pore domain K^+^ channel (K2P) TRAAK (22, 27) and TREK1 (28). The K^+^ channels give rise to leak (also called background) K^+^ currents to stabilize the negative resting membrane potential and counterbalance membrane depolarization [reviewed by (9, 29)]. BK and TRAAK are gated primarily by membrane tension (10, 30–34).

TRPs belong to a superfamily of nonselective cation channels with a higher permeability to Ca^+^ than Na^+^. TRPV1, TRPV2, and TRPV4 may open upon pressure (35), membrane stretch (36), hypotonicity, or fluid flow (37–44), and the expression has been reported in mammalian retinal ganglion cell layer (GCL) (16–18), inner nuclear layer (INL) (20, 45), plexiform layers (15, 17, 18), and bipolar cells (15) in the mouse, rat, cat, and primate retina [reviewed by (46)]. The GCL and INL also express TREK and TRAAK in the mouse retina (27, 28).

MSCs like TRPV2, TRPV4 (37–44), BK, TRAAK (10, 30–34), and ENaC (8) have been established as mechano-gated channels, which are directly gated by force and opened by membrane stretch and pressure. Pioneering studies on retinal mechanical response started from TRPV4 (17) and TRPV1 (14, 16). In these studies, hypotonicity, static pressure, and channel agonists evoked action potentials and calcium currents in mammalian RGCs. The channel opening was associated with RGC apoptosis (14, 16, 17), and TRPV4 antagonists have showed neuroprotective effects on RGC survival *in vitro* (18) and in a rat glaucoma model (47). Our team has recently reported pressure-evoked cation currents in retinal neurons. In primate bipolar cells (BC) (15), dynamic pressure stimuli opened a cation conductance reversed around ~0 mV, which was consistent with the expression of TRPV4. Similarly, in vertebrate retinal rods and cones, dynamic pressure stimuli evoked three membrane currents, which reversed at ~−80 mV or ~0 mV, aligning with the expression of K-MSCs and TRPVs (22). Moreover, a most recent study from our laboratory has observed significant interference of TRPV4 on light responses of photoreceptors and BCs in living mice *in vivo* (23). Functional studies aligning with morphological and genetic observations have generally established the mechanical responsiveness of retinal neurons.

K-MSCs and N-MSCs are responsive to similar mechanical stimuli, but they mediate currents of opposite polarities given the distinctive reversal potential of ~ − 80 mV and ≥ ~0 mV, respectively [reviewed by (46)]. Studies usually focus on the activity or location of individual MSCs but rarely assess the co-expression of multiple MSCs, their relative expression levels, and their combined effects in individual neurons, including retinal neurons (11, 15, 16, 48). Pressure stress is associated with various visual disorders (7, 49–51), but the consequence of pressure-induced activation of retinal MSCs has been unclear.

We investigated the co-presence of several K-MSCs and N-MSCs and the activities of TRPV4 in retinal ganglion cells (RGCs) in the mammalian retina with immunocytochemistry, confocal microscopy, and whole-cell patch-clamp techniques. Our data demonstrated the co-expression of K-and N-MSCs in retinal neurons and TRPV4-mediated RGC dysfunction. The results further suggest that the balance between K-MSCs and N-MSCs, i.e., R_K/N_ ratio, is a core mechanism of cellular mechano-homeostasis and a novel determinant for the pressure vulnerability of individual neurons.

## Methods

### Preparations

All procedures were carried out in strict accordance with the Guide for the Care and Use of Laboratory Animals of the National Institutes of Health, ARVO Statement for the Use of Animals in Ophthalmic and Vision Research, and related regulations of the Institutional Animal Care and Use Committee. The animals were 3-7-month-old male and female C57BL/6J mice purchased from Jackson Laboratory (Bar Harbor, ME, USA). Chemicals were purchased primarily from Sigma-Aldrich (St. Louis, MO, USA) and Tocris Bioscience (Bristol, United Kingdom) except otherwise specified.

### Patch-clamp recording of retinal neurons

Animals were dark-adapted for 1–2 h before recording, and all related procedures were performed under infrared illumination. RGCs were recorded from living whole-mount retinas under the loose patch, voltage-clamp, or current clamp mode. The dual-cell patch-clamp recording used MultiClamp 700A or 700 B amplifiers connected to DigiData 1322a interfaces and operated by pClamp software (Axon Instruments, Foster City, CA, USA). Two EPC10 quadruplet amplifiers with digitizers (HEKA instrument, Holliston, MA, USA) provided eight independent channels, which enabled us to record eight cells simultaneously under either voltage-or current-clamp mode. The recording was performed under infrared illumination (>750 nm) or dim red light and monitored by a Nano video camera (Stemmer Imaging AG, Puchheim, Germany). The novel technology has been recently applied to retinal study for the first time by Pang and colleagues (22, 52, 53).

Electrodes were filled with an internal solution containing 0.1–0.5% Lucifer yellow (LY) and/or 2% neurobiotin (NB) with pH adjusted to 7.3. (54–57). Patch pipettes had 5–8 MΩ tip resistance when filled with an internal solution containing 112 mM Cs-methanesulfonate, 12 mM CsCl, 5 mM EGTA, 0.5 mM CaCl_2_, 4 mM ATP, 0.3 mM GTP, and 10 mM Tris, adjusted to pH 7.3 with CsOH. For current-clamp and some voltage-clamp recordings, the pipettes were filled with internal solutions containing 112 mM K-gluconate, 10 mM KCl, 10 mM EGTA, 10 mM HEPES, 0.5 mM CaCl_2_, 1 mM MgCl_2_, 4 mM Na_2_-ATP, and 0.3 mM Na_3_-GTP, adjusted to pH 7.3 by KOH. The internal solution and the external normal Ringer’s solution yield an E_Cl_ of −59 mV at room temperature. Recorded cells were visualized by LY and/or NB fluorescence with a confocal microscope (LSM 800, Carl Zeiss, Germany).

### Light stimulation

A photostimulator delivered light spots of a diameter of 600–1,200 μm and 500 nm wavelength (λ_max_ = 500 nm, full width-half max 10 nm) at various intensities (−10 to −1 log I) to stimulate the retina via the epi-illuminator of the microscope (55, 56, 58). The intensity of unattenuated [0 in log unit (log I)] 500 nm light from a halogen light source was 4.4 × 10^5^ photons. μm^−2^ sec^−1^. An LED emitting 505 nm light of the unattenuated intensity of 4 × 10^3^ photons.μm^−2^ sec^−1^ will be used for multi-cell patch-clamp recording, delivering light spots of 700–1,200 μm in diameter.

### Immunocytochemistry and retrograde labeling of RGCs

Double-and triple-immuno-labeling followed the published experimental protocols (15, 55, 59–62). All fixed retinas were blocked with 10% donkey serum (Jackson ImmunoResearch, West Grove, PA, USA) in TBS [D-PBS with 0.5% Triton X-100 (Sigma-Aldrich) and 0.1% NaN3 (Sigma-Aldrich)] for 2 h at room temperature or at 4°C overnight to reduce nonspecific labeling. A vibratome (Pelco 102, 1000 Plus; Ted Pella, Inc., Redding, CA, USA) was used to prepare retinal slices. Whole retinas were imbedded in low gel-point agarose (Sigma-Aldrich) and trimmed into a 10 × 10 × 10 mm^3^ block. The block was glued onto a specimen chamber mounted on the vibratome and subsequently cut into 40-mm thick vertical sections in PBS solution. Whole retinas and free-floating sections were incubated in primary antibodies in the presence of 3% donkey serum-TBS for 3–5 days at 4°C. Controls were also processed without primary antibodies. Following several rinses, the slices and whole retinas were then transferred into Cy3-, Cy5-, or Alexa Fluor 488-conjugated streptavidin (1:200, Jackson ImmunoResearch), with Cy3-and/or Cy5-conjugated secondary antibodies (1:200, Jackson ImmunoResearch) and/or Alexa Fluor 488-conjugated secondary antibodies (1:200; Molecular Probes, Eugene, OR), in 3% normal donkey serum-TBS solution at 4°C overnight. A nuclear dye, TO-PRO-3 (0.5 mL/mL, Molecular Probes, Eugene, Oregon, USA) was used with the secondary antibody to visualize the nuclei in the retina. After extensive rinsing, retinal preparations were cover-slipped.

RGCs were identified with a double-retrograde labeling technique previously established by Pang and colleagues (60, 61). Briefly, eyeballs of dark-adapted animals were enucleated under the illumination of dim red light. The nerve stump of the freshly dissected eyeball was dipped into a small drop (3 μL) of 3% Lucifer yellow (Sigma) and/or 8% neurobiotin (NB, Vector Laboratories, CA) in the internal solution (61) for 20 min. Then, the eyeball was thoroughly rinsed with the oxygenated Ames medium (Sigma) to remove the extra dye and dissected under infrared illumination. The dark-adapted eyecup with intact retina and sclera tissue was transferred into fresh oxygenated Ames medium and kept at room temperature for 40 min under a 10 min-dark/10 min-light cycle. Subsequently, the whole retina was isolated from the sclera, fixed in darkness for 30–45 min at room temperature, and visualized with Cy3-, Cy5-, or Alexa Fluor 488-conjugated streptavidin (1,200, Jackson ImmunoResearch). The technique brightly labeled the entire population of RGCs in the mouse retina (60, 61).

### Antibodies and markers

Antibodies were summarized in Table 1. Polyclonal rabbit anti-TRPV4 (LS-C135, 1: 200; LS-A8583 1:200 and LS-C94498 1: 100) was purchased from LifeSpan Biosciences, Inc. (Seatle, WA, USA). LS-C94498 was raised against a synthetic peptide from the cytoplasmic domain (aa100-150) of mouse TRPV4 conjugated to an immunogenic carrier protein. LS-A8583 targets a synthetic 20-amino acid peptide from the internal region of human TRPV4, and LS-C135 was raised against rat TRPV4 (Q9ERZ8, aa853-871, peptide immunogen sequence: CDGHQQGYAPKWRAEDAPL). LS-C135 provided the best signal-to-noise ratio in the primate retina (15). The specificity of LS-A8583 (17), LS-C94498 (17), and LS-C135 (15, 23) for labeling retinal TRPV4 were confirmed in TRPV4 transgenic mice.

**Table 1:** Antibodies.

Name	Source, Catalog number, Host, dilution	Antigen	Specificity	References
TRPV4	Lifespan bioscience, LS-C135, rabbit, 1: 500	Q9ERZ8, aa853-871 of rat TRPV4	Confirmed on mutant mice	(15, 23)
TRPV2 IgG	Calbiochem, PC421, rabbit, 1: 200	Synthetic peptide aa 744–761 of rat TRPV2	Identified a single band at ~90 kDa of the full length TRPV2	(44, 64)
TRPV2	Lifespan bioscience, LS-C112764, goat, 1: 100	Synthetic peptide aa743–756 of mouse TRPV2	Identified a single band at ~100 kDa in rat brain lysates close to MW of 86 kDa of TRPV2	(22)
TRAAK	Santa Cruz, sc-11324, goat, 1: 100	A peptide mapping at the C-terminus of TRAAK of human origin.	Identified a single band at ~47 kDa, matching TRAAK.	(7, 63)
BK (slo 1) monoclonal	Millipore, MABN70, mouse, 1: 500	Recombinant protein corresponding to the S9-S10 segment of mouse slo 1.	Identified a single band at ~100 kDa in rat brain lysates, matching MW 100–130 kDa of BK.	(65, 66)
ENaCα	Abcam, ab65710, rabbit, 1: 200	Amiloride-sensitive ENaCα (human)	Identify a single band at 75 kDa in human platelet lysates, matching ENaCα.	(67–69)
ENaCβ	Abcam, ab21795, mouse, 1: 100	aa 541–640 of Human ENaCβ.	Identified two bands in protein extract of *E. coli* and did not respond to a negative control fusion protein.	
PKCα antiserum	Sigma, P4334, rabbit, 1: 1 K	Synthetic peptide corresponding to aa from the C-terminal V5 region of rat PKCα conjugated to KLH.	Recognized a heavy band at ~76 kDa and a very weak band at 40 kDa in rat brain extract, in line with MW 76–93 kDa of PKCα.	(55, 70, 71)
PKCα monoclonal	BD bioscience, 610107, mouse, 1: 200	Human PKCα aa270-427	a single band at 82 kDa close to MW 76–93 kDa of PKCα.	(55, 70, 71)
Glutamine synthetase (GS) monoclonal	BD Transduction, 610517, 610518, clone 6, mouse, 1: 1 K	human glutamine synthetase aa 1–373	Recognized a band at ~45 kDa, consistent with the predicted MW of GS.	(60)
Calbindin D-28 K	Swant, CB38, rabbit, 1: 1 K	recombinant rat calbindin D-28 K	Immunoblot of brain homogenate of the mouse, rat, guinea pig, rabbit, macaca, zebrafish, and chicken detects only a single band at 28 kDa. Negative results in knockout mice.	(73, 74)
Calbindin-D-28 K, monoclonal	Sigma, C9848, clone CB955, mouse, 1: 200	bovine kidney calbindin-D, 28 kDa	Does not react with other members of the EF-hand family	(73, 74)

Purified polyclonal goat-anti TRAAK was purchased from Santa Cruz (sc-11324, goat, 1: 100). It was raised against a peptide mapping at the C-terminus of TRAAK of human origin, which identified a single band at ~47 kDa in IMR-32 and Y79 whole cell lysates, matching TRAAK (63). TRPV2 antibodies included a rabbit anti-rat (1: 200, PC241, Calbiochem, San Diego, CA, USA) and a goat anti-mouse (LSbio, Lynnwood, WA, USA) polyclonal antibody, and the antigens were a synthetic peptide aa 744–761 of rat TRPV2 (44, 64) and a peptide aa743-756 of mouse TRPV2 with the sequence C-SEEDHLPLQVLQSH. Monoclonal mouse anti-BK was purchased from MilliporeSigma (1: 500, MABN70, anti-Slo1, clone L6/60, pore-forming alpha unit), and it primarily identifies a single band ~100 kDa close to MW 100–130 kDa of BK (65, 66). Polyclonal rabbit anti-ENaCα was from Abcam (1, 200, Cambridge, MA, USA) (67–69), which identified a single band at ~75 kDa in human platelet lysates, matching ENaCα.

Protein Kinase-C alpha (PKCα) is a classic marker for rod BCs (55, 70, 71). The anti-PKCα antibody from Sigma (P4334, 1: 1000, rabbit, polyclonal) was tested in immunoblotting with rat brain extract, and it recognized a heavy band at ~76 kDa and a very weak band at 40 kDa, while the predicted molecular weight of the PKCα was 76–93 kDa. The staining was specifically inhibited by PKCα immunizing peptide (659–672). The monoclonal anti-PKCα antibody from BD transduction [610107, Clone 3/PKCα (RUO), 1: 200, mouse] identified a single band at 82 kDa from a rat cerebrum lysate. Two calbindin D-28 k antibodies were used to identify horizontal cells (72), one of which was a rabbit polyclonal antibody raised against the recombinant rat calbindin D-28 K (CB) protein purchased from Swant (Marly 1, Switzerland) (CB38, 1: 1000) (73, 74), and the other was a mouse monoclonal antibody purchased from Sigma and produced with the bovine kidney calbindin-D (C9848, clone CB955, 1: 200) (73, 74). Monoclonal mouse anti-glutamine synthetase (GS) (1: 1000, clone 6, BD Transduction Laboratories, Palo Alto, CA) was used to identify Mȕller cells (60). The antibody was raised against the human glutamine synthetase aa 1–373 and recognized a band at ~45 kDa, consistent with the predicted molecular weight of GS. Other primary antibodies included in this study have also been used in previous reports, including polyclonal guinea pig anti-GABA (1:1000, AB175; Chemicon, Temecula, CA, USA) (75) and rat anti-glycine antiserum (1:1000, a generous gift from Dr. David Pow, University of Queensland, Brisbane, QLD, Australia) (76). The specificity of these primary antibodies has been demonstrated in previous studies, and their staining patterns in our results were similar to the reports. Controls were also processed with blocking peptides or without primary antibodies. All controls did not show positive results.

### Data analysis

We used confocal microscopes (510 and LSM 800, Carl Zeiss, Germany) for morphological observation. Pixel intensities of MSC immunoreactivities were analyzed in different retinal layers with the confocal (Zen Blue, Zeiss), Sigma plot (v12 and 15), and Excel software and fitted to an exponential function (15).


(1) $$f\left(I\right)=a*\exp\left\{-0.5*{\left[\frac{\left(I-I_{0}\right)}{b}\right]}^{2}\right\}$$

where *a* is the pixel count at the distribution peak, *I* is the pixel intensity, and *I_0_* is *I* at the distribution peak, and *b* is a slope/width factor. The ATP consumed by RGCs for maintaining resting membrane potential (ATP_RP_, molecules/s) was calculated by the previously established equation (95)


(2) $$ATP_{RP}=\frac{N_{A}}{F}*\frac{\left(V_{Na}-RP\right)*\left(RP-V_{K}\right)}{R_{m}*\left(RP+2*V_{Na}-3*V_{K}\right)}$$

where *V_Na_* (+50 mV) and *V_K_* (−100 mV) are Nernst potentials for Na^+^ and K^+^, *N_A_/F* = 6.2 × 10^18^, and *N_A_ and F* are the Avogadro and Faraday constant, respectively. Data were further analyzed by Sigmaplot (v12 and v15, Systat, Point Richmond, CA, USA), Clampfit (v10.3 and v9.2, Axon Instruments, Foster City, CA, USA), Matlab, and Microsoft Excel for statistical significance. The *α* level to reject the null hypothesis is 0.05.

## Results

### BK expression in retinal layers

**Figure 1:**
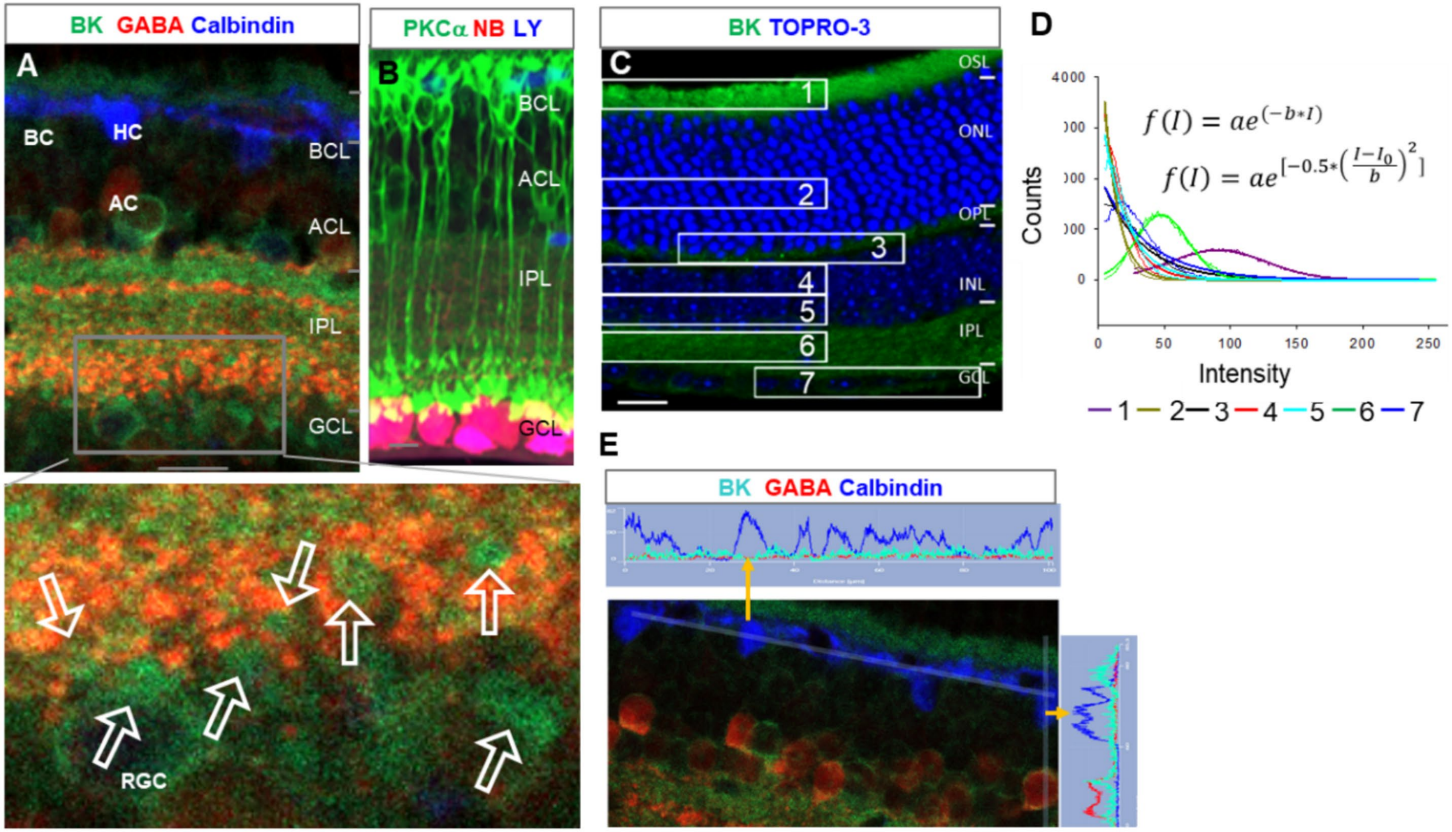
BK expression in the mouse retina. **(A,E)** Retinal slices labeled for BK (green), GABA, and Calbindin. **(B)** The retina was retrogradely labeled for RGCs with Lucifer yellow (LY, blue) and neurobiotin (NB, red) and stained for PKCα (green), showing the size and location of the characteristic globules of axon terminals of rod bipolar cells (RBCs). **(C)** Labeled for BK (green) and TO-PRO-3. **(D)** The fitting curves and functions of pixels of BK immunoreactivity (IR) in seven retinal layers in **(C)**. The BK-IR is primarily present in the OSL and IPL. Weak BK-IR is present in some somas in the INL and GCL and large globules of RBCs (arrows, inset of **A**), which contact BK-and GABA-positive profiles of putative A17 ACs. **(E)** The two plots show the linear profile of pixels along the two straight lines in the image. HCs identified by calbindin were nearly negative for BK. BCs, ACs, HCs, and RGCs: bipolar, amacrine, horizontal, and ganglion cells, respectively. OSL: photoreceptor layer. ONL and INL: inner and outer nuclear layer, respectively. OPL and IPL: inner and outer plexiform layer, respectively. Scale bars are 20 μm.

BK was the most heavily expressed in the outer segment layer (OSL) (Figures 1C,D), and weaker immunoreactivity (IR) was also present in the OPL, IPL, some somas in the INL and GCL (Figure 1A), and large globules of axon terminals of rod bipolar cells (RBCs) (Figure 1B and the inset of Figure 1A). Horizontal cells identified by calbindin D-28 K antibody were nearly negative for BK (Figure 1E). The peak intensity of the fitting curve of the pixel histogram (Equation 1) showed that the relative intensity of BK-IR was in the order of *OSL > IPL≈RBC axons > OPL≈GCLINL.* BK-IR identified some GABAergic somas of ACs and dendrites of putative A17 ACs that contacted the axon terminal of PKCα-positive RBCs (Figure 1A).

### TRAAK expression in retinal layers

**Figure 2:**
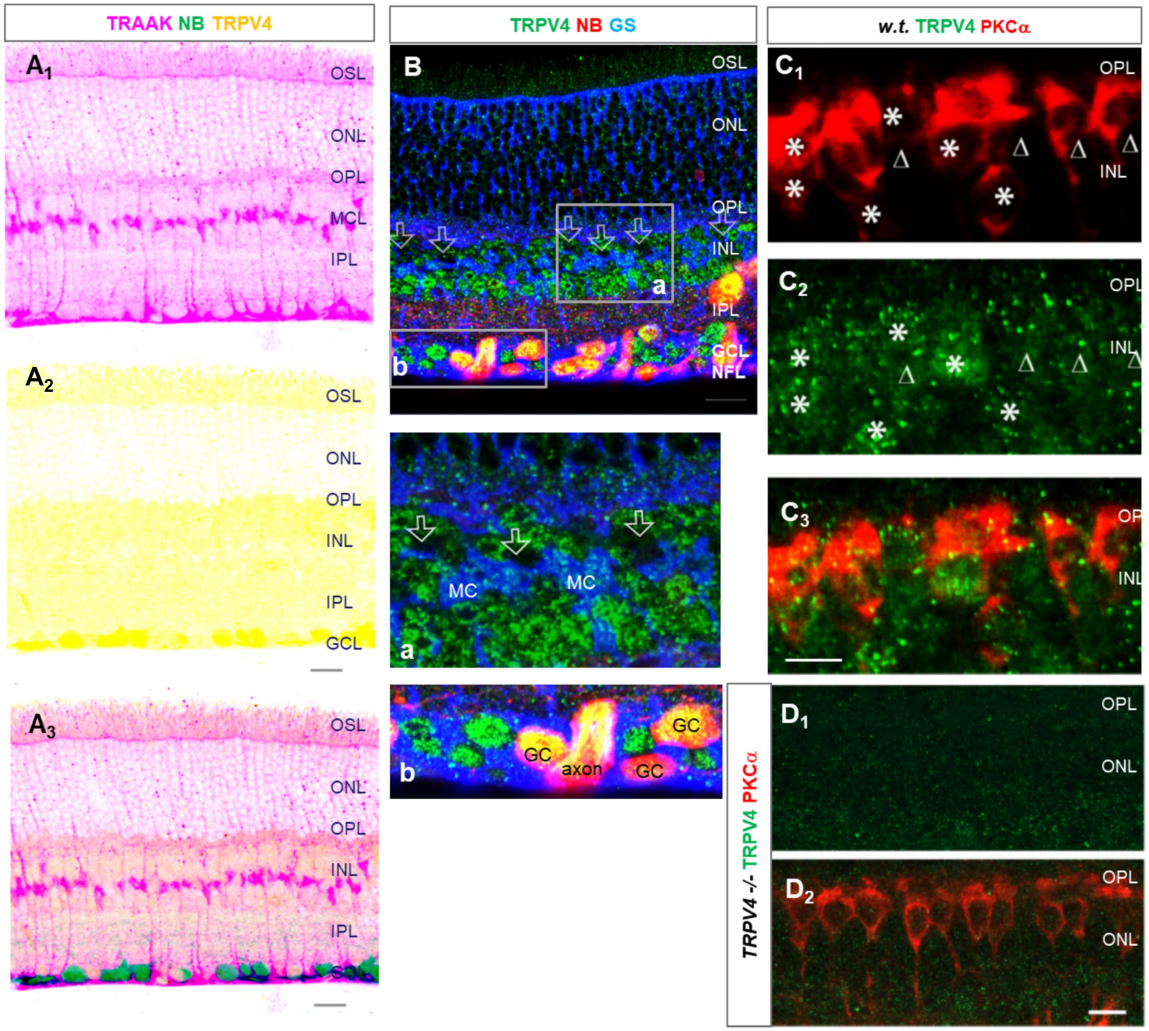
The expression of TRAAK and TRPV4 in the mouse retina. **(A,B)** Retinal slices were retrogradely labeled for ganglion cells (GCs) by neurobiotin (NB) and stained for TRPV4 (green) and TRAAK (**A**, pink) or glutamine synthetase (GS, blue, **B**). TRPV4 signals are heavier in the GCL. **(A)** The image was inverted and displaced on a white background. TRAAK is heavily expressed in Müller cells and weakly expressed in OSL and OPL. **(B)** Axons and somas of GCs are brightly positive for TRPV4. TRPV4 is present in Müller cells (MC). Some somas of putative cone BCs in the second soma row of the INL contain no TRPV4 signals (open arrow, inset b). **(C,D)** Retinal slices from wild-type (w.t.) and TRPV4 transgenic mice (TRPV4−/−) were labeled for TRPV4 (green) and PKCa (red). **(C)** Some TRPV4 puncta are present in rod BCs (asterisks) and cone BCs (triangles). **(D)** TRPV4 signal is nearly absent in TRPV4 transgenic mice. OSL: outer segment layer; ISL: inner segment layer; OPL: outer plexiform layer; INL: inner nuclear layer; BCL-bipolar cell layer; ACL-amacrine cell layer; IPL-inner plexiform layer; GCL-ganglion cell layer; NFL-nerve fiber layer. The scale bar is 5 μm for C and 20 μm for others.

In triple-labeled retinal slices, TRAAK (Figure 2A_1_) was heavily expressed in Müller cells, including the somas in the middle of the INL, end feet in the GCL, and descending processes passing the INL and IPL. The OSL and OPL were weakly labeled. The relative intensity of TRAAK-IR was in the order of *Müller cells > OSL≈ OPL*. In Figures 2A,B, RGCs were retrogradely identified, and RGC somas were nearly negative for TRAAK.

### The co-expression of depolarizing TRPV4 with the hyperpolarizing BK and TRAAK

In labeled retinal slices, TRPV4-IR was more intensive in retrograde-labeled RGC somas in the GCL (Figures 2A_2_,B). It was weaker in other layers and nearly absent in the inner segment layer (ISL) of photoreceptors. TRPV4-IR was mostly diminished in homozygotes with TRPV4 expression suppressed (*TRPV4^−/−^* mice), demonstrating the specificity of the antibody (Figure 2D). TRPV4-IR was also present in PKCα-identified RBCs (Figure 2C, asterisk), some PKCα-negative cone bipolar cells (Figure 2C, triangle), and GS-identified Mȕllar cells (Figure 2B). Some somas of putative cone bipolar cells were negative for TRPV4 (Figure 2B, inset b). The relative intensity of TRPV4-IR was in the order of *GCL > OSL≈ OPL≈ IPL≈ INL*.

The results (Figures 1, 2) together revealed that the OSL expressed BK, TRAAK, and TRPV4; RGCs possessed the highest level of TRPV4 and a low level of BK and lacked TRAAK; INL, IPL, and OPL weakly co-expressed TRPV4 and BK. The higher level of depolarizing TRPV4 in RGCs is consistent with their higher pressure-vulnerability observed in glaucoma (1, 2) and traumatic retinal injury (3–6) compared to other neurons.

### The expression of depolarizing TRPV2 and ENaC in the mouse retina

**Figure 3:**
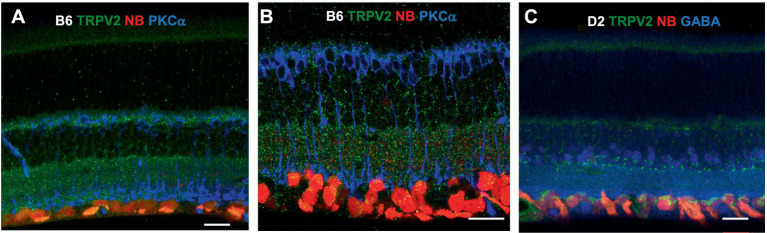
TRPV2 expression in mouse retina. RGCs were retrogradely labeled by neurobiotin (NB, red). **(A,B)** TRPV2 (green) is expressed in the OSL, OPL, IPL, and GCL **(A)**. Some puncta are present in RBCs identified by PKCα immunoreactivity (**B**, blue). The expression in the ACL and GCL is heavier in DBA/2J mice (D2, a congenital glaucoma model, in **C**), including amacrine cells labeled for GABA (**C**, blue). B6: C57BL/6J mice. Scale bars are 20 μm.

TRPV2 was expressed primarily in the OPL, IPL, and retrograde-identified RGCs, and TRPV2-IR was weak in the OSL and INL and nearly absent in the ISL. The relative intensity of TRPV2-IR was in the order of *IPL≈ OPL > GCL > OSL≈ INL* for wild-type mice. TRPV2-IR was heavier in the GCL and ACL in the congenital glaucoma model DBA/2J (D2) mice, and the relative intensity was in the order of *GCL≈ ACL > OPL≈OSL* (Figure 3).

**Figure 4:**
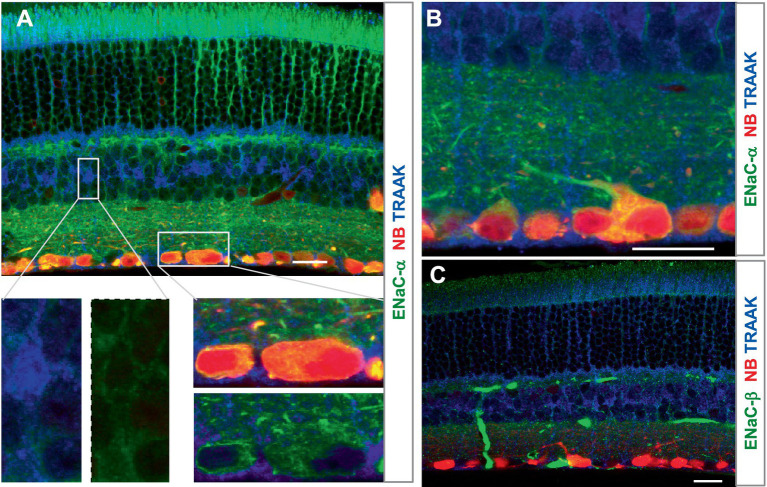
The expression of the mechanosensitive ENaC in mouse retina. Retinas were retrogradely labeled for RGCs by NB (red) and stained for TRAAK (blue) and ENaCα **(A,B)** or ENaCβ **(C)** (green). **(A,B)** ENaCα is intensively expressed in photoreceptors, OPL, IPL, and RGC somas and dendrites, and the INL is weakly labeled. TRAAK is primarily expressed in the soma, process, and end foot of Müller cells and OSL **(A–C)**. **(C)** ENaCβ identifies blood vessels and weakly labels the OSL, OPL, and IPL. Scale bars are 20 μm.

ENaCα was intensively expressed in photoreceptors, OPL, IPL, and RGC somas and dendrites, and the INL was weakly labeled. The relative ENaCα-IR was in the order of *photoreceptors≈ GCL≈ OPL≈ IPL > INL* (Figure 4). The ENaCβ antibody stained blood vessels and weakly labeled the OSL, OPL, and IPL (Figure 4C). The distribution of these depolarizing MSCs was nearly even in the outer and inner retina but favored neurons over Müller cells. In Figures 3, 4, all RGCs were retrogradely identified.

In summary, the relative expression level was in the order of *Müller cells > OSL≈ OPL* for TRAAK, *OSL > IPL≈ RBC axons > OPL≈ GCL≈ INL* for BK, *GCL > OSL≈ OPL≈ IPL≈ INL* for TRPV4, *GCL≈ ACL > OPL≈ OSL* for TRPV2 (in D2 mice), and *GCL≈ photoreceptors≈ OPL≈ IPL > INL* for ENaCα. TRAAK was expressed mainly in Müller cells. Photoreceptors expressed BK and ENaCα intensively and TRAAK, TRPV2, and TRPV4 weakly. RGC somas and axons retrograde-identified clearly expressed ENaCα, TRPV4, and TRPV2 but lacked TRAAK and BK. BCs weakly expressed BK, ENaCα, and TRPV4. Some amacrine cells were positive for BK, ENaCα, and TRPV4. These results indicate a higher ratio of K-MSCs to N-MSCs (R_K/N_) in photoreceptors and interneurons than RGCs, which is consistent with the high pressure-vulnerability of RGCs in glaucoma.

### TRPV4-induced excitation and dysfunction of mouse RGCs

**Figure 5:**
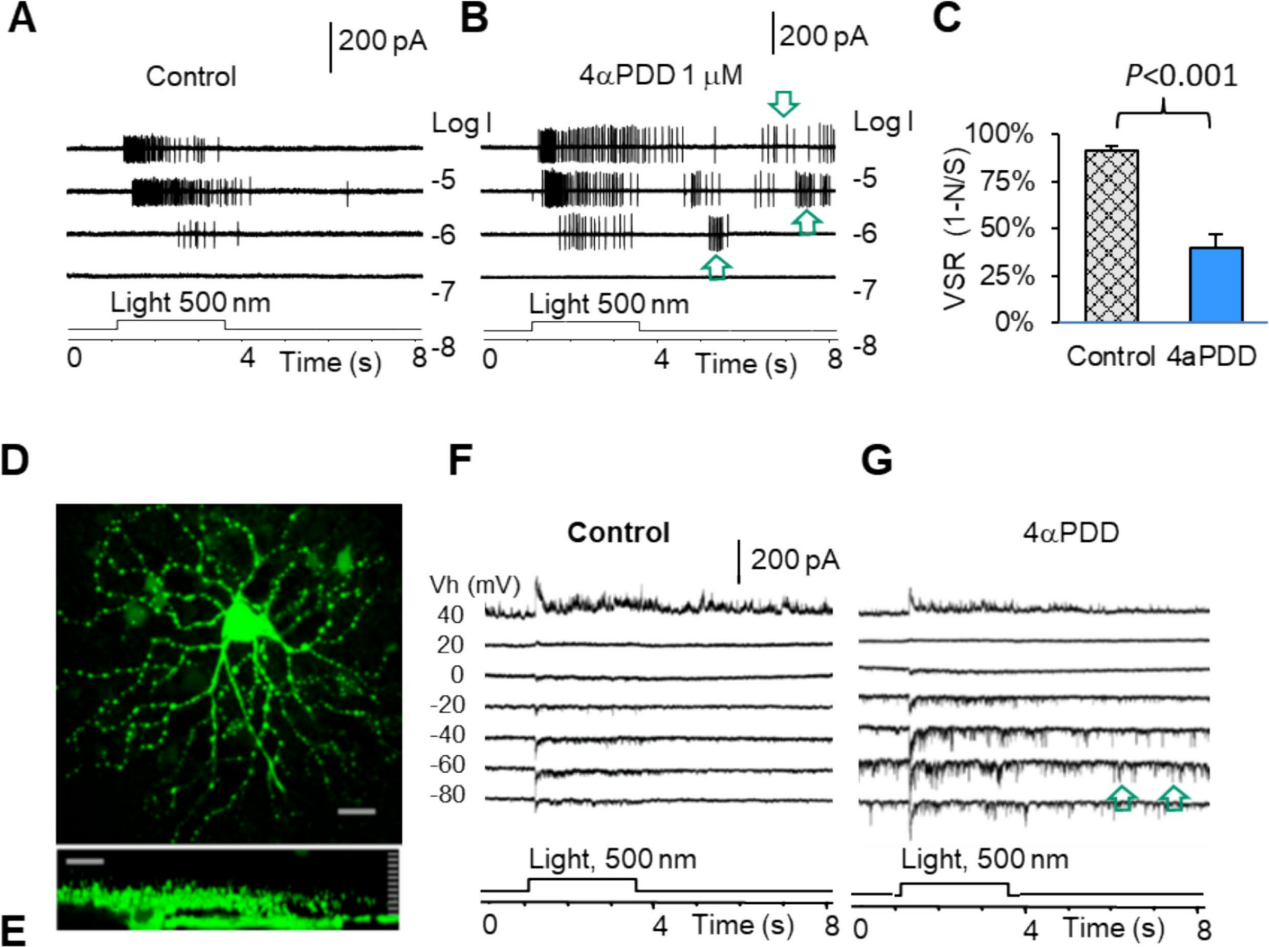
TRPV4 mediates excitatory visual noises and dysfunction of mouse RGCs. RGCs were recorded under loose patch mode **(A,B)** and whole-cell voltage-clamp mode at various holding potentials **(D–G)**. **(A,B,F,G)** TRPV4 agonists enhance spontaneous action potentials (**A,B**, arrow) and the spontaneous postsynaptic currents (arrow) and light-evoked excitatory currents **(F,G)**, reducing the visual signal reliability [VSR = 1-noise (N)/signal (S)] by ~50% **(C)**. **(D,E)** Confocal images of a recorded RGC filled with Lucifer yellow (**D**: The x-y view; **E**: The y-z view revealing the dendritic tree and axon of the cell).

We have previously characterized mouse RGCs by the resting potential, synaptic currents, and firing features (57, 77). Since TRPV4 mediates excitatory cation current, we further study the role of TRPV4 in RGCs with TRPV4 agonists 4α-Phorbol 12,13-didecanoate (4αPDD) and GSK101 (GSK, GSK1016790A). The results showed that TRPV4 agonists enhanced the firing rate of spontaneous action potentials (Figures 5A,B) recorded under the loose-patch mode and the frequency and amplitude of the spontaneous and light-evoked excitatory postsynaptic currents (sEPSCs and ΔI_C_, respectively) (Figures 5D–G) under the voltage-clamp mode in all RGCs. In Figures 5A,B, the ONαRGC generated light-evoked action potentials (APs) with little visual noise, while 4αPDD induced many spontaneous APs, i.e., visual noises, which were unrelated to the light. Similarly, Figures 5F,G showed that in another ONαRGC, 4αPDD enhanced the amplitude and frequency of sEPSCs unrelated to the light. We further measured the frequency of light-evoked APs (Signal, S) and light-unrelated APs (Noise, N) in the same RGCs before and after application of drugs. In the presence of the drugs, the visual signal reliability (VSR), calculated by (VSR = 1-N/S) (Figure 5C), was reduced by ~50% (*p* < 0.001) in ON RGCs. Since the versicle release from presynaptic terminals primarily determines the frequency of sEPCSs and the postsynaptic mechanism dominates the amplitude of sEPSCs, these data indicate that the TRPV4-induced excitation in RGCs involves TRPV4 in both BCs and RGCs.

**Figure 6:**
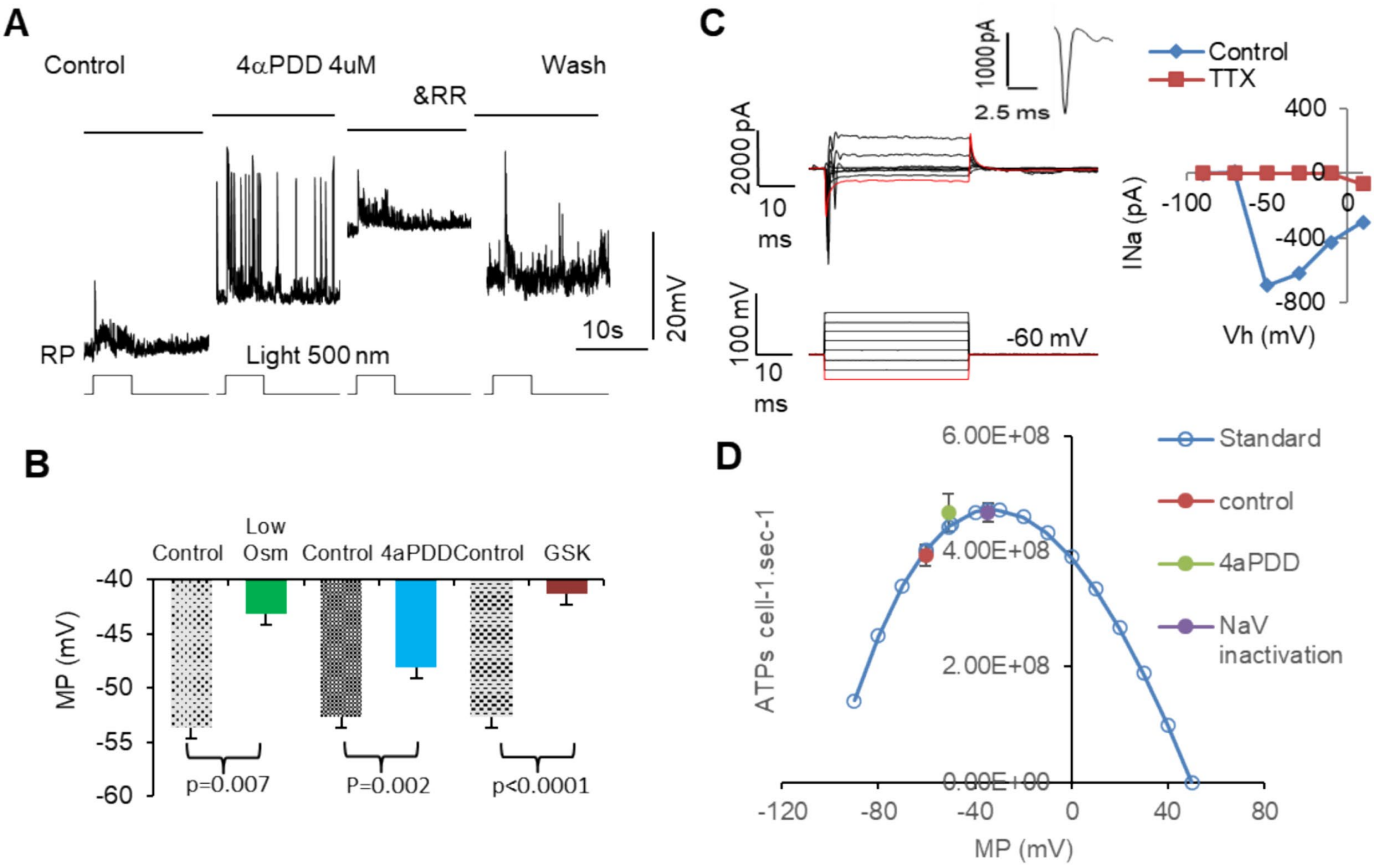
The activation of MSCs induces membrane depolarization, ATP depletion, and dysfunction of mouse RGCs. RGCs are recorded under current-clamp **(A,B)** and voltage-clamp **(C)** modes. **(A,B)** Activating MSCs by low osmolarity (Osm) and TRPV4 agonists 4aPDD and GSK101 (GSK) depolarizes MP of RGCs to −40 to −50 mV and elicits spontaneous firing of action potentials **(A)**, which raises the ATP consume close to the peak level and predicts ATP depletion. **(C)** The threshold of NaVs in mouse RGCs is close to −50 mV and sensitive to TTX. The duration of the sodium current is ~2 ms. **(D)** Blue curve: the standard ATP consumption for RGCs to maintain RP was plotted per Equation 2. TRPV4 agonists raised the ATP consumption (green dot) near the inactivation level of half NaVs (purple dot). Vh: holding potential. MP: membrane potential. RP: resting potential. NaV: voltage-gated sodium channel. RR: ruthenium red, nonspecific blocker of TRPs.

Figure 6 showed that activating MSCs with low osmolarity (Osm) and TRPV4 agonists 4αPDD and GSK101 significantly depolarized membrane potential in all RGCs (Figures 6A,B). 4αPDD elicited spontaneous firing of APs (Figure 6A) was reversibly blocked by a general TRPV antagonist ruthenium red (RR). TRPV4 induced membrane depolarization and spontaneous firing would need to consume extra ATPs. Thus, we plotted the ATP consumption for maintaining normal resting potential based on Equation 2 (Figure 6D, blue curve). The data showed that TRPV4-induced depolarization raised the ATP consumption to the peak, predicting ATP depletion. We also recorded Na^+^ currents (I_Na_) in RGCs mediated by voltage-gated Na^+^ channels (NaV) under voltage-clamp conditions by depolarizing RGCs from −110 or − 70 mV with a step of 8–20 mV. The evoked responses could not be significantly affected by BC and AC synapses. I_Na_ was an inward current at the holding potentials of ≤40 mV and identifiable from potassium, chlorides, and calcium currents by the duration of ~2 ms and the polarity of the spike. I_Na_ was found to activated at ~ − 50 mV (*n* = 5 cells) and sensitive to TTX (Figure 6C), consistent with typical NaVs well documented in previous studies (78, 79). The peak of the ATP consumption curve just overlapped with the membrane potential (~ − 37 mV) where half NaVs underwent slow inactivation (purple dot, Figure 6D) (80), and TRPV4 agonists also raised the ATP consumption to the peak level (green dot, Figure 6D), indicating that ATP depletion and RGC malfunction may follow intensive TRPV4 activation.

The results together demonstrated that TRPV4 activation directly induced depolarization and dysfunction of RGCs, and the effect is associated with TRPV4 expressed in BCs and RGCs, expedited inactivation of NaVs in RGCs, and ATP overconsumption.

## Discussion

### Individual retinal neurons co-express several K-and N-MSCs for cellular mechano-electro-homeostasis

The retina expresses multiple MSCs (11, 21, 46), while studies often focus on individual types of MSCs. It has been unclear whether individual retinal neurons co-express multiple MSCs and the significance of such design. The elevation in intra-and extra-ocular pressure closely involves the pathogenesis of glaucoma and other retinal conditions (1–7), and the question of whether retinal neurons were directly responsive to the mechanical signals had not been addressed until recent years when mechanical responses were revealed from RGCs (14, 17), BCs, and photoreceptors (15, 22). MSCs generate electric currents in retinal neurons upon activation. Since many MSCs, including TRPV4, TRPV2, BK, TRAAK, and ENaCa, are not classic molecules that serve light pathways in the retina, their physiological and pathophysiological role remains to be defined (11, 46, 81–84).

We discovered the co-expression of several MSCs in retinal layers and individual neurons, including N-MSCs that mediate inward cation currents and K-MSCs that carry outward K+ current at membrane potential levels between −80 and 0 mV (46). The morphological data, consistent with functional results obtained from photoreceptors and BCs in the salamander (22) and mammalian retina (15, 23), demonstrates that outer retinal neurons possess several types of MSCs. We postulate that a cell with more MSCs would theoretically possess better mechanical adaptability, and because of the opposite polarity of the electric currents at K-and N-MSCs, a balanced ratio of K-and N-MSCs would better reduce the pressure-induced instantaneous electric disturbance on neuronal signals. Supporting this idea, our data revealed a distinct R_K/N_ ratio in retinal neurons, and the more balanced one in the outer retinal neurons well aligns with their lower pressure vulnerability in glaucoma and traumatic retinal injury (1–7). It indicates that the pressure responsiveness of a cell is to be determined by both the level and ratio of K-and N-MSCs, establishing a novel mechanism of cellular mechanical homeostasis in sensory neurons that may provide not only mechano-protection to cellular physical integrity but also electro-protection to neuronal signals by reducing mechanically induced electric noises.

Recent work has also identified the interaction between TRPs and BK in smooth muscles and between TRPV4 and another calcium-activated potassium channel KCa2.3 in the endothelial cells of blood vessels (85–87). BK mediates muscle relaxation, and TRPV4 mediates contraction after the relaxation (86), supporting the notion of the counterbalance between these channels.

### The relative level of hyperpolarizing MSCs and depolarizing MSC is critically associated with neuroprotection and neurodegeneration

RGCs showed higher pressure vulnerability in glaucoma and traumatic retinal injury than other retinal neurons (1–7). Depolarizing N-MSCs such as TRPV4 and TRPV1 can mediate RGC depolarization, spontaneous firing, and cell death (14, 15, 17, 88), and our patch-clamp data was in line with previous reports. More importantly, we used VSR to demonstrate TRPV4-mediated RGC dysfunction, and we further calculated TRPV4-related ATP depletion and the likelihood of inactivating voltage-gated sodium channels (NaVs). Meanwhile, our immunological data confirmed a much higher expression level of depolarizing N-MSCs relative to K-MSCs in RGCs but similar levels of N-and K-MSCs in outer retinal neurons. These results together support the idea that a low *R_K/N_* or a high *R_N/K_* plays a pro-neurodegenerative role in neurons.

The excitotoxic effect of TRPVs previously reported in RGCs has suggested that blockage or removal of TRPVs may be beneficial (14, 15, 17, 18, 88), but whether it would compromise the pressure adaptability of the neurons is still uncertain. ENaC and TRPVs carry cation currents to reverse at ≥ ~0 mV, while K-MSCs like BK and TRAAK mediate background leak currents to reverse at ~ −80 mV (46). Thus, at the membrane potential (RP) level of 0 > RP > −80 mV, K-MSCs would counterbalance N-MSC-induced membrane depolarization. Based on current results, raising *R_K/N_* appears to be a safer strategy to reduce pressure-induced excitotoxicity than solely reducing N-MSCs.

Membrane potential alters the driving force of MSCs and is another factor that modulates currents at MSCs besides *R_K/N_*, while RGCs maintain RPs less depolarized than outer retinal neurons (22, 57, 89–93). Hence, we postulate that RGCs are less protected by K-MSCs and more easily activated by N-MSCs compared to outer retinal neurons, and this mechanism could also contribute to the high vulnerability of RGCs. Our data discovered a low R_K/N_ in RGCs, suggesting *R_K/N_* as a novel determinant of pressure-induced excitotoxicity and improving R_K/N_ as a new therapeutic strategy.

### MSCs mediate electric noise, dysfunction, and ATP crisis in RGCs

RGCs express TRPV4 in the mouse (16, 17, 94), porcine (18), and primate retina (15), and TRPV4 agonists excite RGCs (15, 17). Our data are consistent with previous findings. Besides membrane depolarization, we revealed that TRPV4 agonists caused more robust excitatory postsynaptic current (EPSC_TRPV4_) and dramatic reduction of VSR, predicting ATP depletion in RGCs. EPSC_TRPV4_ may involve TRPV4-IR in both BCs and RGCs.

Using VSR (1-N/S, %), we measured the reliability of the light-evoked action potentials under the disturbance of TRPV4 opening, creating an accurate measure for the dysfunction of individual RGCs. Neurons consume most energy to maintain the normal membrane potential (i.e., the normal electrochemical gradient of ions), as the latter is frequently disrupted by the opening of ion channels on the plasma membrane (95). Cation fluxes via opened ion channels follow and, thus, consume their electrochemical gradients across the membrane. The reduced gradients need ATPase and energy to recover (95). We first explored the relationship between the effect of TRPV4 activation, RP, activities of NaVs, and ATP consumption in RGCs. Our results showed that TRPV4 activation depolarized RGCs to the RP level that would inactivate NaVs (78–80, 96) and maximize ATP consumption. The results further suggest that improving ATP levels should be considered in treating pressure-related visual diseases.

Although currents at K-and N-MSCs are generally mutually compensating because of the opposite polarity, TRPs are known to mediate transient currents (15, 43, 84, 97), and K-MSCs carry leak currents (22, 28, 29). The difference in their kinetics does not allow complete cancelation of the pressure-induced current noise even with a balanced R_K/N_. Our data indicates that opening TRPVs in retinal neurons can reduce VSR and increase ATP consumption in RGCs, predicting ATP crisis and pathologies if the pressure stress is persistent. Further studies on MSCs in retinal neurons will likely substantially facilitate the early diagnosis and treatment of pressure-related retinal disorders.

## Data Availability

The original contributions presented in the study are included in the article/supplementary material, further inquiries can be directed to the corresponding author.
